# A Structure-Guided Mutation in the Major Capsid Protein Retargets BK Polyomavirus

**DOI:** 10.1371/journal.ppat.1003688

**Published:** 2013-10-10

**Authors:** Ursula Neu, Stacy-ann A. Allen, Bärbel S. Blaum, Yan Liu, Martin Frank, Angelina S. Palma, Luisa J. Ströh, Ten Feizi, Thomas Peters, Walter J. Atwood, Thilo Stehle

**Affiliations:** 1 Interfaculty Institute of Biochemistry, University of Tübingen, Tübingen, Germany; 2 Department of Molecular Biology, Cell Biology and Biochemistry, Brown University, Providence, Rhode Island, United States of America; 3 Department of Chemistry, University of Luebeck, Luebeck, Germany; 4 Glycosciences Laboratory, Faculty of Medicine, Imperial College London, London, United Kingdom; 5 Biognos AB, Gothenburg, Sweden; 6 Department of Pediatrics, Vanderbilt University School of Medicine, Nashville, Tennessee, United States of America; Fred Hutchinson Cancer Research Center, United States of America

## Abstract

Viruses within a family often vary in their cellular tropism and pathogenicity. In many cases, these variations are due to viruses switching their specificity from one cell surface receptor to another. The structural requirements that underlie such receptor switching are not well understood especially for carbohydrate-binding viruses, as methods capable of structure-specificity studies are only relatively recently being developed for carbohydrates. We have characterized the receptor specificity, structure and infectivity of the human polyomavirus BKPyV, the causative agent of polyomavirus-associated nephropathy, and uncover a molecular switch for binding different carbohydrate receptors. We show that the b-series gangliosides GD3, GD2, GD1b and GT1b all can serve as receptors for BKPyV. The crystal structure of the BKPyV capsid protein VP1 in complex with GD3 reveals contacts with two sialic acid moieties in the receptor, providing a basis for the observed specificity. Comparison with the structure of simian virus 40 (SV40) VP1 bound to ganglioside GM1 identifies the amino acid at position 68 as a determinant of specificity. Mutation of this residue from lysine in BKPyV to serine in SV40 switches the receptor specificity of BKPyV from GD3 to GM1 both *in vitro* and in cell culture. Our findings highlight the plasticity of viral receptor binding sites and form a template to retarget viruses to different receptors and cell types.

## Author Summary

Viruses need to bind to receptors on host cells for viral entry and infection, and the type of receptor bound determines the range of hosts and tissues the virus can infect. Viruses within a family often vary in their tissue distribution and pathogenicity because changes in receptor specificity can produce a virus with different spread and infectivity. In fact, many transmissions between species are based on a virus acquiring binding capability for a new receptor. The structural changes that underlie such receptor switching are not well understood. We have analyzed the structural requirements for receptor binding and switching of the human BK polyomavirus (BKPyV), the causative agent of polyomavirus-associated nephropathy. We show that BKPyV uses specific gangliosides that all contain a common α2,8-disialic acid motif to infect cells, and have characterized the interaction in atomic detail. Our data explains the requirement for this disialic acid motif and in particular highlights a single amino acid that is central to determining specificity. Mutation of this residue switches the receptor specificity, enabling BKPyV to infect cells bearing a different class of gangliosides. Our findings highlight the plasticity of viral receptor binding sites and form a template to retarget viruses to different receptors and cell types.

## Introduction

Interactions of a virus with receptors on host cells are crucial for viral entry and infection, and determine host range and tissue tropism of the virus. As a result, receptor specificity and affinity are tightly regulated, with changes in either producing a virus with different spread and infectivity. Many zoonotic transmissions are based on a virus acquiring binding capability for a new receptor. Despite their importance, most viral specificity switches are poorly characterized, especially for carbohydrate-binding viruses, as methods capable of structure-specificity studies are only being developed for carbohydrates. Here we characterize the receptor specificity of the human BK Polyomavirus in depth, and retarget it to use the Simian Virus 40 (SV40) receptor GM1.

The human polyomavirus BK Virus (BKPyV) is a non-enveloped, double-stranded DNA (dsDNA) virus that belongs to the family *Polyomaviridae*. Other members of the family include Simian Virus 40 (SV40), the human JC Virus (JCPyV), Merkel Cell Polyomavirus (MCPyV) and at least eight other recently discovered human polyomaviruses [1]. BKPyV was first isolated from a kidney transplant recipient in 1971 [2]. It establishes a persistent asymptomatic infection in the genitourinary tract of approximately 70% of the adult population [3], [4], [5]. A key modulator of BKPyV reactivation is immunosuppression of the host that leads to an increase in viral replication [3]. Complications of BKPyV reactivation include the development of polyomavirus-induced nephropathy (PVN) in kidney transplant recipients, and hemorrhagic cystitis in bone marrow transplant recipients [3], [6], [7].

**Figure 1:**
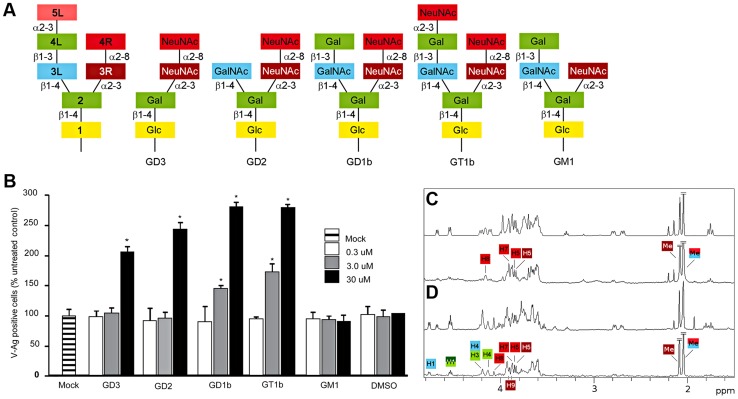
B-series gangliosides are receptors for BKPyV. (A) Schematic representation of structures of disialic acid-containing b-series gangliosides GD3, GD2, GD1b, GT1b and of the monosialylated a-series ganglioside GM1 indicating the abbreviated designations used here for individual residues in the gangliosides. (B) Ganglioside supplementation assays. Vero cells were incubated with gangliosides, challenged with BKPyV and scored for infection. The average number of VP1 positive cells is plotted compared to controls. Error bars represent the standard deviation for 3 independent experiments. Asterisks indicate p-value (*p<0.05). (C) STD off-resonance (top) and difference (bottom) spectra of WT BKPyV VP1 in the presence of 50-fold excess GD3 oligosaccharide. The off-resonance spectrum was scaled to 3%. GD3 resonances labeled in the difference spectrum receive considerable saturation transfer from the protein. Regions with strong signal overlap are not labeled because saturation effects in this region cannot be unambiguously assigned. Signals that were truncated are denoted by diagonal bars. (D) STD off-resonance (top) and difference spectrum (bottom) of wild type BKPyV in the presence of 50-fold excess GD1b oligosaccharide. The spectrum is labeled as in C.

BKPyV attachment is mediated by cell-surface sialic acid [8]. The most common sialic acid type in humans is 5-*N*-acetyl neuraminic acid (NeuNAc) [9]. Simians and most other mammals, however, possess an enzyme that can attach an additional hydroxyl group to NeuNAc, yielding 5-*N*-glycolyl neuraminic acid (NeuNGc). In contrast to humans, these animals therefore carry both NeuNAc and NeuNGc. Gangliosides were found to mediate cell attachment of BKPyV [10], and gangliosides GD1b and GT1b were later shown to function as specific receptors for BKPyV [11] (Fig. 1). Gangliosides are ceramide-based glycolipids, which are used as receptors for most of the well-characterized polyomaviruses, for example GD1a and GT1b for Polyoma, or GM1 for SV40 [12].

A polyomavirus capsid consists of 72 pentamers of the major capsid protein VP1 [13], [14]. Crystal structures of Polyoma, SV40, MCPyV and JCPyV VP1 show that receptors are bound in shallow grooves formed by VP1 loop structures on the outer surface of the virus. These loops contribute to different receptor specificities and are the only parts of VP1 that are not well conserved [14], [15], [16], [17], [18].

In this study, we use viral infection assays to demonstrate that the b-series gangliosides GD3, GD2, GD1b and GT1b enhance BKPyV infection. We then define the common α2,8-disialic acid motif on these gangliosides as the primary binding epitope for BKPyV by NMR spectroscopy. In order to understand how the disialic acid motif is recognized by the virus, we solved the crystal structure of a BKPyV VP1 pentamer in complex with GD3 and generated a model of the pentamer with the larger GD1b oligosaccharide. Analysis of these complexes reveals extensive interaction with the terminal sialic acid, specificity-defining contacts with the internal sialic acid, thus explaining the requirement for a disialic acid motif, and additional contacts with the branched GD1b. Mutagenesis of residues contacting the disialic acid motif abolishes infectivity and a comparison with the SV40 VP1-GM1 complex attributes the different viral receptor specificities to one point mutation. Introduction of this mutation into BKPyV switches specificity, enabling BKPyV to bind GM1 and abolishes binding to GD3, as shown by saturation transfer difference (STD) NMR spectroscopy and carbohydrate microarray analyses. The microarray analyses moreover reveal that the mutant is specific for the ‘human’ sialic acid NeuNAc. This contrasts with SV40 VP1, which has a preference for the more prevalent NeuNGc found in simian species and many other nonhuman mammals. The specificity of the mutant thus sheds light on the influence of sialic acid on species tropism.

## Results

### All b-series gangliosides support infection of BKPyV

To date, only four gangliosides had been tested to support BKPyV infection, two of which, the b-series gangliosides GD1b and GT1b, were confirmed as receptors [11]. The carbohydrate moieties of gangliosides typically consist of two “arms” (Fig. 1A). In this manuscript, we number the carbohydrates sequentially (starting from the lipid anchor) and use “L” and “R” designations to indicate whether a carbohydrate is part of the left or the right arm, respectively. For example, NeuNAc 3R is the third carbohydrate and located in the right arm (Fig. 1A, schematic on the left hand side). The right arm consists of only sialic acids and is used to classify gangliosides. The b-series gangliosides, e.g. GD1b and GT1b, carry both NeuNAc 3R and NeuNAc 4R, while a-series gangliosides such as GM1 carry only NeuNAc 3R (Fig. 1A). We tested the effects of all common b-series gangliosides on BKPyV infection by supplementing permissive Vero cells with GD3, GD2, GD1b or GT1b (Fig. 1B). Consistent with previous reports [11], gangliosides GD1b and GT1b enhanced infectivity of Vero cells. However, gangliosides GD2 and GD3, which had not been tested previously, also enhanced infection of the cells (Fig. 1B). Incorporation of all b-series gangliosides into the plasma membrane of Vero cells increased the binding of labeled VP1 pentamers to cells (data not shown). In control experiments, supplementing cells with the a-series ganglioside GM1 had no effect on infection or binding (Fig. 1B, and data not shown). The ability of b-series gangliosides to enhance BKPyV infection was greater in the presence of the left arm, with GD1b and GT1b supporting infection best (Fig. 1B). Taken together, these data show that the α2,8-disialic acid motif in b-series gangliosides is the minimal requirement for binding, with the left arm of GD1b and GT1b contributing some additional interactions.

### BKPyV carbohydrate epitope mapping on b-series gangliosides

We analyzed pentamer binding to GD3 and GD1b oligosaccharides by STD NMR spectroscopy [19], which identifies ligand atoms that contact a protein in solution. The strongest saturation transfer from BKPyV VP1 to GD3 was observed for the methyl group of the terminal NeuNAc 4R, followed by the methyl group of the internal NeuNAc 3R (Fig. 1C). No significant transfer was observed to any of the anomeric protons, or to the NeuNAc H3 protons. Interestingly, no transfer was observed for the Glc and Gal residues of GD3 (Fig. 1C), suggesting that they may not participate in binding.

We repeated the same experiment for GD1b oligosaccharide, observing transfer to essentially the same set of protons from the disialyl moiety plus additional transfer to Gal 4L and GalNAc 3L in the GD1b left arm as well as the branching Gal 2 residue (Fig. 1D). Resonances H4 and H1 from Gal 2 and H1 from GalNAc 3L can be unambiguously assigned, but H3 from Gal 2 and H4 from GalNAc 3L overlap and cannot be distinguished. From the Gal 4L ring, only the anomeric proton can be assigned in the STD difference spectrum. The STD spectra thus show that while the right arms of both GD3 and GD1b interact with BKPyV VP1 in a similar way, additional contacts are provided by the left arm of GD1b.

### Structure of a BKPyV VP1-GD3 complex

**Figure 2:**
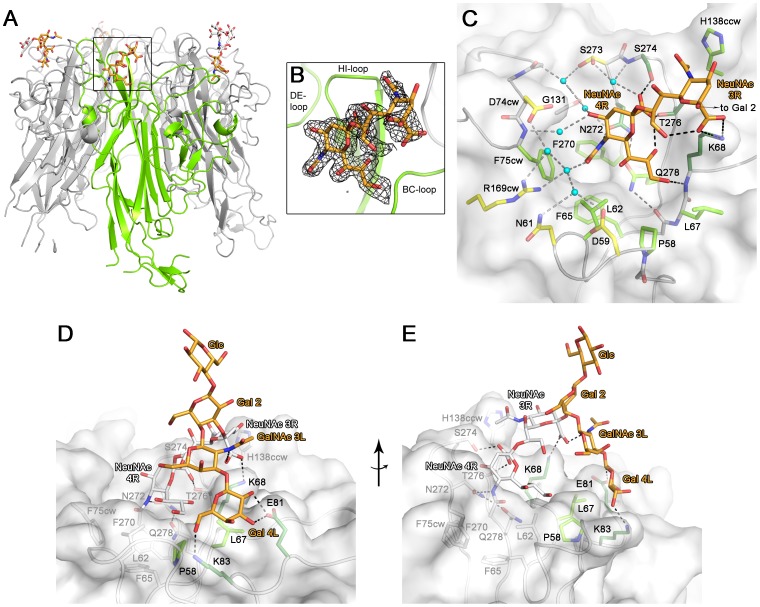
Structure of a BKPyV VP1-GD3 oligosaccharide complex. (A) Structure of BKPyV VP1 pentamer in complex with GD3 oligosaccharide. One VP1 monomer is highlighted in green. Monosaccharides unbiased by crystal contacts are colored orange, while the ones binding to crystal contacts are colored white. (B) Composite annealed difference electron density for the terminal disialic acid motif of GD3 oligosaccharide bound to BKPyV VP1 at a σ level of 2.5. cw/ccw = belonging to the clockwise/counterclockwise neighboring VP1 monomer within the pentamer. (C) Interactions of GD3 oligosaccharide with BKPyV VP1. The oligosaccharide is shown in orange. Side chains contacting the sugar are colored as follows: those making hydrogen bonds are colored dark green, those making van der Waals interactions are light green, and those making water-mediated hydrogen bonds are colored yellow. Atoms of the protein backbone are shown in gray, and water molecules are in cyan. Direct hydrogen bonds are indicated as black dashed lines, water-mediated ones are grey. (D+E) Model of the interaction of BKPyV VP1 with GD1b oligosaccharide. The left arm and the stem of GD1b are colored orange. Residues interacting with the left arm of GD1b are colored as in (C). The disialic acid motif of GD1b and the protein residues contacting it are colored white.

To define the structural features underlying the receptor-binding specificity of BKPyV, we solved the structure of the BKPyV VP1 pentamer at 2.0 Å resolution (Table 1). The VP1 pentamer is a doughnut-shaped ring, with the five monomers arranged around a central pore that aligns with the five-fold symmetry axis (Fig. 2A). The monomers adopt a β-sandwich fold with jelly-roll topology that is present in many viral capsid proteins. The β-strands B, I, D, G and C, H, E, F (designated alphabetically from the N-terminus of the full-length protein) are linked by extensive loops that decorate the surface of the protein. For clarity, the long BC-loop is subdivided into loops BC1 and BC2, which face in different directions.

**Table 1:** Crystallographic data collection and refinement statistics.

	BKPyV VP1		BKPyV VP1 - GD3 complex	
**Data collection**				
Space group	P2_1_2_1_2		P2_1_2_1_2	
Unit cell [Å]	144.9, 152.3, 62.7		144.7, 152.6, 63.2	
Resolution [Å]	50 - 2.0	2.05 - 2.00	50 - 1.7	1.74 - 1.70
Total reflections	388,470	27,741	1,103,641	64,273
Unique reflections	94,342	6,826	154,169	11,266
R_meas_ [%]	12.7	67.0	7.1	69.4
Completeness [%]	99.8	98.4	100.0	99.8
I/σI	11.01	2.40	19.44	2.66
**Refinement**				
R_work_ [%]	16.1	22.0	15.0	23.1
R_free_ [%]*	19.0	25.1	17.7	24.9
Protein atoms	9,957		10,181	
Average B-factor [Å^2^]	23.1		21.5	
Carbohydrate atoms	-		197	
Average B-factor [Å^2^]	-		33.1	
Solvent atoms	899		1,327	
Average B-factor [Å^2^]	30.0		32.6	
r.m.s.d.				
Bond lengths [Å]	0.010		0.010	
Bond angles [°]	1.246		1.311	
B-factor Wilson [Å^2^]	27.1		26.4	

r.m.s.d. = root-mean-square deviation.

* R_free_ was calculated with 3.5% of the data.

We incubated BKPyV VP1 crystals in 20 mM GD3 oligosaccharide solution and solved the structure of the resulting complex at 1.7 Å resolution (Table 1). Attempts to form a complex with GD1b oligosaccharide by soaking or co-crystallization failed in several crystal forms, likely because of the size of the GD1b hexasaccharide. However, the GD3 structure encompasses the minimal motif required for binding and provides a template for understanding interactions with the larger GD1b oligosaccharide. The structures of unliganded and liganded BKPyV VP1 are virtually identical (r.m.s.d. of 0.4 Å for all atoms of one monomer), indicating that GD3 binding does not induce a conformational change in the protein. GD3 engages BKPyV VP1 at the top of the pentamer, which corresponds to the outer surface of the virion (Fig. 2A, B). Contacts involve residues in the BC1-, HI- and DE-loops of one monomer, as well as the BC2-loop of the clockwise neighboring VP1 monomer (BC2cw) and the DE-loop of the counterclockwise neighbor (DEccw). Four of the five binding sites within one pentamer are occupied by ligand, while one is inaccessible due to crystal packing. Two of the four occupied binding sites, however, participate in crystal contacts. As their conformations are influenced by these non-physiologic interactions, they will not be considered further. The two remaining GD3 oligosaccharides do not participate in crystal contacts and assume an identical conformation, which therefore should represent a physiologically relevant complex. In both cases, only the terminal NeuNAc-α2,8-NeuNAc motif interacts with BKPyV VP1, while the Gal-Glc moiety projects into solution. This is in agreement with the STD NMR data that showed little saturation transfer between BKPyV VP1 and the Gal-Glc portion of GD3.

### Contacts between BKPyV VP1 and GD3

The terminal NeuNAc 4R is the main contact of GD3 with BKPyV VP1. In all four occupied binding sites on the BKPyV VP1 pentamer, NeuNAc 4R adopts the same conformation and makes identical interactions with the protein. The sialic acid carboxylate group is recognized by two hydrogen bonds to the side chains of S274 and T276 (Fig. 2C). Additional, water-mediated hydrogen bonds are formed to the side chain of S273 and the backbone of S274. The O4 hydroxyl group of NeuNAc 4R interacts via water-mediated hydrogen bonds with the side chain of N272, the backbone of G131, and the backbone nitrogen of F75cw. The *N*-acetyl group makes a hydrogen bond to N272 and a water-mediated hydrogen bond to R169cw. Its methyl group inserts into a tight-fitting, hydrophobic pocket on BKPyV VP1 that is formed by four non-polar residues (L62, F65, F270 and F75cw). Finally, the glycerol chain of NeuNAc lies in a shallow groove and makes van der Waals contacts to the side chains of P58, L62, L67, K68 and Q278. The glycerol chain also forms a single hydrogen bond to the K68 backbone nitrogen. The conformation of the NeuNAc 4R ring is stabilized by an intramolecular hydrogen bond from the carboxylate group to the O8 hydroxyl group.

The second sialic acid of GD3, NeuNAc 3R, has weaker electron density and makes fewer contacts with the protein (Fig. 2C). Its carboxylate group forms a salt bridge with the K68 side chain. The methyl group of its *N*-acetyl chain stacks against a hydrophobic surface created by parts of the side chains of H138ccw, S274 and T276. The prominent role of van der Waals interactions with the methyl group mirrors the NMR results, which feature prominent saturation transfer to the methyl groups of both NeuNAcs (Fig. 1C).

### Modelling of the BKPyV-GD1b interaction reveals additional contacts

The BKV-GD3 complex structure enabled us to model the interaction of BKV VP1 with the longer GD1b oligosaccharide, using the tightly bound terminal NeuNAc 4R as an anchor. A large number of possible GD1b oligosaccharide conformations was calculated and superposed on the terminal NeuNAc 4R in the BKV-GD3 complex structure. The oligosaccharide conformations were filtered for presence of the specificity-defining contacts between the protein and the internal NeuNAc 3R. The remaining conformations could be classified into two groups: one in which the left arm of GD1b pointed away from the protein, not engaging in interactions, and one in which this arm made additional contacts with BKV VP1. Some of the latter conformations were then subjected to a final round of molecular dynamics (MD) simulations in explicit water. We found that the ‘left’ arm is involved in several weaker interactions with amino acids P58, D59, L67, K68, E81, and K83. A snapshot of the complex is shown in (Fig. 2D,E). According to this model, the Gal 4L residue of GD1b can adopt a position enabling hydrogen bonds between its 2- and 3-hydroxyl groups and the side chain of E81, as well as hydrogen bonds between its 6-hydroxyl group and the side chain of K83 and the backbone carbonyl of P58. Moreover, the left arm of GD1b is supported by van der Waals interactions with the side chain of L67, and there is an intramolecular hydrogen bond between the carboxylate group of the internal NeuNAc 3R and the *N*-acetyl group of GalNAc 3L. The model is in accord with the observed increase in BKV infection with increasing length of the left arm of b-series gangliosides, and also with the STD NMR data that show signals for some protons in the left arm.

### Carbohydrate binding is crucial for BKPyV spread and infectivity

**Figure 3:**
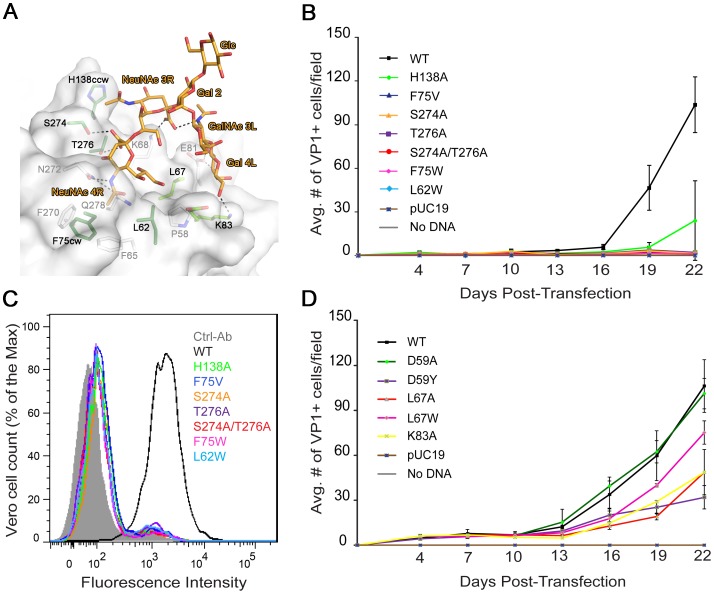
Mutation of residues that interact with the left and right branches of GD1b abolish or reduce growth. (A) Structural model of the BKPyV-GD1b complex. Residues targeted for mutation are colored dark green for the disialic acid motif and light green for the left arm binding sites, respectively. (B) Growth assay for the sialic acid binding site mutants. Vero cells were transfected with linearized WT or mutant BKPyV DNA. Cells were fixed permeabilized and stained for VP1 at 3 day intervals for 22 days and analyzed by indirect immunofluorescence. Viral spread was quantified by scoring for cells expressing VP1. The average number of VP1 positive cells is plotted from 3 independent experiments with each time point representing the average number of infected cells per visual field for 8 fields. (C) Binding of the sialic acid binding site mutants to Vero cells. Cells were incubated with purified His-tagged WT or mutant BKPyV pentamers and an Alexa Fluor 488 conjugated penta-His secondary antibody. Cells were fixed and pentamer binding to cells assessed by flow cytometry. Histograms show the fluorescence intensity of the Alexa 488 antibody alone (gray-filled), WT pentamer (black) and mutants (color) for 1×10^4^ events. (D) Growth assay for the left arm binding site mutants assessed as in B.

To test the biological relevance of these interactions mutations were introduced into an infectious clone of BKPyV. We first probed the interaction with the tightly bound terminal NeuNAc 4R (Fig. 3A) with mutations designed to abolish carbohydrate binding either by reducing the number of hydrogen bonds (S274A, T276A, and S274A/T276A), eliminating van der Waals contacts (F75V), or by introducing steric hindrance (L62W, F75W). Vero cells were transfected with mutant or wild-type (WT) BKPyV plasmid DNA. Viral gene expression was scored every 3 days over a 22 day growth period. The first data point after transfection indicated no difference between wild-type and the mutants in terms of protein expression and localization (data not shown). While WT BKPyV resulted in viral production that continued to spread with time, all mutants that targeted the binding site for terminal sialic acid did not propagate, highlighting the importance of these interactions (Fig. 3B). We then targeted the binding site of the internal NeuNAc 3R. Mutant H138A, in which a van der Waals contact is removed from the second sialic acid, propagated at a significantly reduced level compared to WT (Fig. 3B). We also introduced the mutations in our recombinant pentamer construct. Flow cytometry binding assays using WT and mutant pentamers show that the mutants have greatly reduced cell binding (Fig. 3C), suggesting that the loss of viral propagation is due to an attachment defect. The structural integrity of mutant pentamers was verified with circular dichroism spectroscopy, and their ability to assemble into pentamers was confirmed with gel filtration (data not shown). All the mutations were in the receptor binding site, which is distant from the sites important for capsid assembly. Thus, the mutations are very unlikely to cause defects in capsid assembly.

Finally, we mutated residues in the putative binding site for the left arm of b-series gangliosides (Fig. 3D). Again, we designed mutations to either introduce steric hindrance (D59Y and L67W) or to remove contacts (D59A, L67A and K83A). The mutations that created steric hindrance significantly reduced BKPyV spread in culture. The D59A mutation had no significant effect, but L67A and K83A both reduced BKPyV growth. In addition, the E81A mutant, which also removes a contact from the second arm, was described in an earlier paper to have slightly reduced growth [20]. The mutations likely did not abolish growth altogether because they still permit interactions with the primary contact, terminal sialic acid. While the D59Y and L67 mutations might in theory also interfere with primary sialic acid binding due to the branched nature of GD1b, the K83A and E81A mutations certainly only target the second arm. Thus, mutagenesis confirms our structural model and highlights the importance of specific contacts with the second arm of GD1b. Taken together, our biological data confirm that the binding site for terminal sialic acid is indispensable for viral infection, while peripheral interactions further enhance binding and infection.

### Structural basis of specificity

BKPyV is most closely related to SV40 and JCPyV, with amino acid identity among their VP1 proteins as high as 74%. Nevertheless, the three viruses recognize different sialic acid containing receptors. BKPyV interacts with α2,8-linked b-series gangliosides, while SV40 binds the branched α2,3-linked GM1 ganglioside [12] and JCPyV attaches to the linear α2,6-linked sequence in LSTc [17].

**Figure 4:**
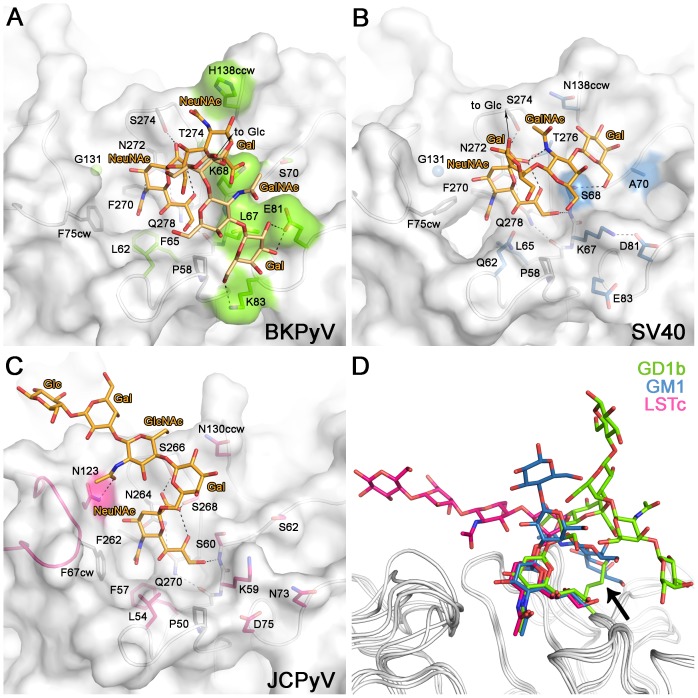
Carbohydrate binding sites of BKPyV, JCPyV and SV40. Recognition of carbohydrate receptors by BKPyV (A), SV40 (B), and JCPyV (C). Only residues making direct hydrogen bonds or van der Waals interactions are shown. Residues that make conserved interactions are colored in yellow, side chains whose positions are not conserved are shown in green (BKPyV), blue (SV40) and pink (JCPyV). (D) Comparison of carbohydrate ligands of BKPyV, SV40 and JCPyV. The oligosaccharides are colored green, blue and pink for the BKPyV, SV40 and JCPyV ligands, respectively. The structures were aligned using the protein main chain.

In receptor complexes of all three viruses, the terminal sialic acid engages in critical and highly conserved interactions that anchor the ligand to VP1 (Fig. 4) [16], [17]. Specificity for the three different oligosaccharide receptor sequences arises in each case from a small number of unique contacts outside the sialic acid binding site. JCPyV recognizes an L-shaped conformation of the LSTc oligosaccharide. The key residue that makes contacts to both legs of this L-shaped glycan is N123 [17] (Fig.4C). BKPyV (Fig. 4A) and SV40 (Fig. 4B) both have a glycine at the equivalent position and thus cannot form similar contacts. BKPyV VP1 specificity for the α2,8-disialyl motif can be attributed to residues K68 and H138, which form contacts with the internal NeuNAc 3R. These two residues are not conserved in SV40 or JCPyV, and thus neither virus is able to specifically interact with α2,8-disialic acid carrying glycans in the same manner [16], [17]. Moreover, none of the BKPyV residues contacting the left arm of gangliosides are conserved in either SV40 or JCPyV. Although the SV40 receptor, GM1, resembles GD1b with an identical left arm, BKPyV and SV40 VP1 bind the left arm at different sites on the proteins (Fig. 4A,B). The two pockets recognize the left arm in different ways. The GalNAc 3L methyl group of GM1 in the SV40 complex is bound at a similar position on VP1 as the NeuNAc 3R methyl group in the BKPyV complex. BKPyV cannot bind the left arm of GM1 in the orientation seen in the SV40 complex because the binding site is blocked by the large side chain of K68, which would lead to clashes with GalNAc (Fig. 4A,B). Apart from this difference, BKPyV and SV40 VP1 share similar surface features at the SV40 left arm binding site and display the same main chain conformation in their surface loops. Thus, the inability of BKPyV to bind to GM1 appears to be determined by the amino acid at position 68.

### A single point mutation enables BKPyV to recognize GM1

**Figure 5:**
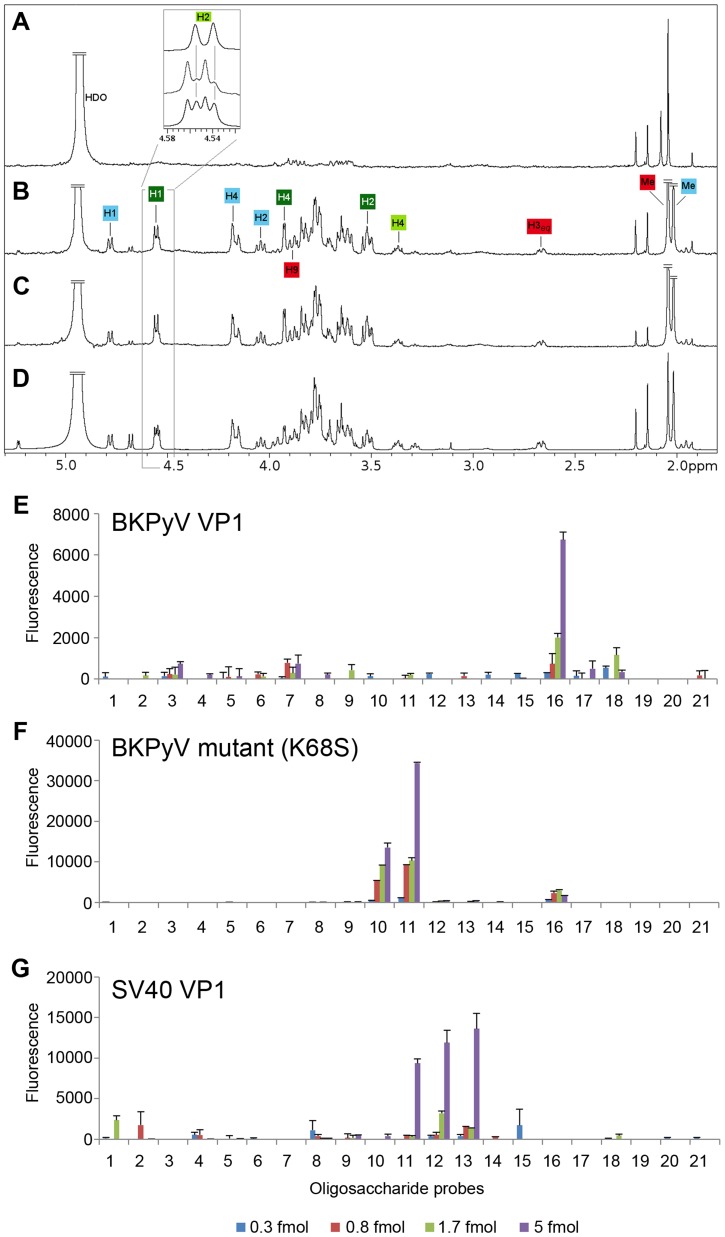
The K68S mutation targets BKPyV to the SV40 receptor GM1. STD difference spectra of (A) BKPyV K68S with GD3, (B and C, respectively) BKPyV K68S and SV40 with GM1. (D) SV40-GM1 off-resonance spectrum. A 50-fold excess of oligosaccharide was used for each spectrum. The off-resonance spectrum was scaled to 3%. Resonances labeled in the difference spectra with GM1 (B and C) receive considerable saturation transfer from BKPyV K68S and SV40. Regions with strong signal overlap are not labeled because they cannot be unambiguously assigned. Binding of BKPyV K68S to GD3, previously seen for WT BKPyV (Fig. 1C), is abolished by the mutation (A). Carbohydrate microarray analyses of recombinant VP1 of (E) BKPyV, (F) BKPyV K68S and (G) SV40 using 21 ganglioside-related saccharide probes, which included the b-series gangliosides as well as GM1 variants NeuNAc-GM1 and NeuNGc-GM1. The doses of probes arrayed per spot are indicated. Numerical scores of the binding signals are means of duplicate spots (with error bars). The complete list of probes and their sequences are in Supplemental Table S1.

To validate the conclusions derived from the structural comparisons, we introduced a K68S mutation into the BKPyV VP1 pentamer expression construct. Purified K68S pentamers were analyzed by STD NMR for binding to GD3 and GM1. Unlike the WT BKPyV-GD3 pair, almost no saturation transfer was observed for BKPyV K68S and GD3, indicating that the mutation virtually abolished binding to the disialic acid motif of GD3 (Fig. 5A). However, saturation transfer from BKPyV K68S VP1 to GM1 was as efficient as for the SV40 VP1-GM1 pair, which was included for comparison (Fig. 5B–D). This indicates that the K68S mutation switches the binding preference of BKPyV VP1 from GD3 to GM1. The STD NMR spectra of SV40 VP1 and BKPyV K68S VP1 with GM1 are almost indistinguishable, suggesting that GM1 engages in the same contacts with both proteins. Saturation transfer is primarily observed to protons of the NeuNAc 3R and Gal 4L rings. In addition, both the GalNAc and the NeuNAc methyl groups in GM1 received considerable saturation in the complexes, with the NeuNAc methyl group being more affected. Our observations are in good agreement with the crystal structure of the SV40 VP1-GM1 complex [16] and demonstrate that a single amino acid mutation suffices for BKPyV to adapt to the SV40 receptor.

### BKPyV K68S mutant is specific for the human sialic acid NeuNAc

BKPyV, JCPyV and SV40 differ in one aspect of their sialic acid binding site. The cavity engaging the methyl group is tight-fitting and lined with hydrophobic residues in BKPyV and JCPyV, but significantly enlarged and partially hydrophilic in SV40 (Fig. 4A–C). This difference may reflect the different hosts of these viruses, humans and simians, and the different types of sialic acids characteristic for each host. While the most prominent sialic acid in humans is NeuNAc, simians carry in addition to NeuNAc larger amounts of *N*-glycolyl neuraminic acid (NeuNGc), in which the methyl group is replaced by the bigger and more hydrophilic glycolyl (CH_2_-OH) group [9], [21]. SV40 preferentially binds to NeuNGc-GM1, and the glycolyl group likely engages polar residues in the cavity [16], [22]. By contrast, the smaller and more hydrophobic cavity of BKPyV and JCPyV cannot accommodate the glycolyl group in a similar manner, thus making BKPyV and JCPyV specific for NeuNAc.

To assess their specificity for human-type and simian-type sialic acids, the VP1 proteins of WT BKPyV, mutant BKPyV K68S, and SV40 were analyzed using a focused ganglioside microarray comprised of 21 ganglioside-related saccharide probes, which included the b-series gangliosides as well as GM1 variants NeuNAc-GM1 and NeuNGc-GM1 (Supplemental Table S1). Microarray analyses revealed differing binding specificities of the three VP1 proteins (Fig. 5E–G). With the WT BKPyV, the only detectable binding was to the b-series ganglioside GD1b (position 16) and the signal intensity was relatively low. No binding to any other b-series gangliosides GD3, GD2, GT1b or GQ1b was detected, possibly due the lower binding avidity to these probes compared to GD1b in the array (Fig. 5E). The BKPyV K68S mutant showed barely detectable binding to GD1b, but highly specific and strong binding to the two NeuNAc-GM1 probes (positions 10 and 11), which differed only in the composition of their lipid moieties (Fig. 5F). Interestingly, there was no binding to the simian-type NeuNGc-GM1 probes, in contrast to SV40 VP1, which showed preferential binding to the two NeuNGc-GM1 probes (positions 12 and 13) (Fig. 5G). This finding is in accord with earlier observations and consistent with our structural analysis (Fig. 4).

### BKPyV K68S uses NeuNAc-GM1 as a receptor on human cells

**Figure 6:**
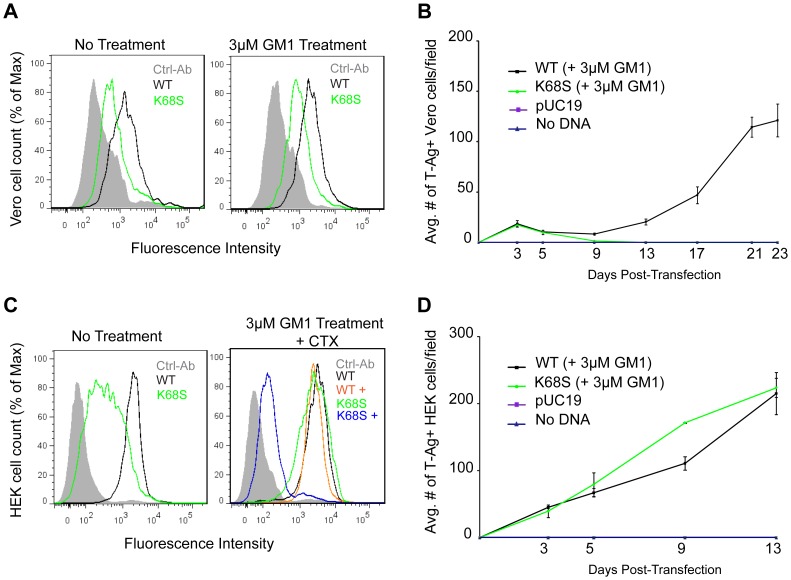
K68S BKPyV uses NeuAc-GM1 as a receptor for attachment and infection. (A) Binding of BKPyV K68S to Vero cells. Cells were treated as in 3C fixed and pentamer binding to cells untreated (left) or treated with NeuNAc-GM1 (right) assessed by flow cytometry. Histograms show the fluorescence intensity of the Alexa 488 antibody alone (gray-filled), WT pentamer (black) and K68S pentamer (green) for 1×10^4^ events. (B) Growth assay for BKPyV K68S in Vero cells. Cells were transfected as previously described, treated with NeuNAc-GM1, fixed and stained over 23 days. Viral spread was quantified by scoring for cells expressing T-Ag. The anti-V antigen monoclonal antibody 597 used in Figures 1 and 3 recognizes an epitope that is disrupted by the K68S mutation, requiring the use of an mAb against T-Ag. The average number of T-Ag positive cells is plotted from 3 independent experiments. (C) Binding of BKPyV K68S to HEK cells. Cells were treated as previously described, fixed and pentamer binding to cells untreated (left), or treated with NeuNAc-GM1 and CTX (right) assessed by flow cytometry. Histograms show the fluorescence intensity of the Alexa 488 antibody alone (gray-filled), K68S pentamer (green) WT pentamer (black), WT pentamer with CTX (orange) and K68S pentamer with CTX (blue) for 1×10^4^ events. (D) Growth assay for BKPyV K68S in HEK cells. Cells were transfected as previously described, treated with NeuNAc-GM1, fixed and stained over 13 days. Viral spread was quantified as above. Gangliosides were added every 3 days.

We next tested whether BKPyV K68S was able to use human-type NeuNAc-GM1 to attach to cells. Purified K68S or WT BKPyV VP1 pentamers were incubated with simian (Vero) and human (HEK) cells, and binding was detected by flow cytometry. In both Vero and HEK cells, K68S mutant pentamers had reduced binding compared to WT pentamers, reflecting either lower affinity or a lower number of receptor molecules (Fig. 6A,C). There was no significant change in the binding of K68S or WT VP1 pentamers to Vero cells that were supplemented with 3 µM NeuNAc-GM1 prior to incubation with the pentamers (Fig. 6A). However, binding of K68S VP1 to HEK cells was increased upon supplementation with GM1, whereas WT binding levels were unchanged (Fig. 6C). This finding might be linked to the enzyme CMP-sialic acid hydroxylase, which converts NeuNAc to NeuNGc and is present on simian but not human cells [9]. We performed a competitive binding assay with GM1 treated HEK cells in the presence and absence of Cholera toxin subunit B (CTX), which uses GM1 as a receptor [23]. CTX abolished binding of K68S VP1, confirming that the K68S mutant is in fact retargeted to GM1 (Fig. 6C). CTX had no effect on the binding of WT BKPyV VP1 (Fig. 6C).

The K68S mutation was also assayed in long-term viral growth assays. We found that while transfection of WT BKPyV plasmid into Vero cells resulted in viral propagation and spread, transfection with the K68S plasmid failed to propagate (Fig. 6B). In human cells however the K68S mutant spread as efficiently as WT regardless of supplementation with GM1 (Fig. 6D).

## Discussion

In this structure-function study, we investigated the interaction of BKPyV with its glycan receptors and identified key determinants of specificity. We show that the conserved α2,8-disialic acid motif on the right arm of b-series gangliosides is the minimal binding epitope for BKPyV, with the variable left arm contributing some additional contacts. Point mutations in the receptor binding site abolish viral spread and infectivity, demonstrating the physiological relevance of the observed interactions.

Our data demonstrate that all of the b-series gangliosides tested support BKPyV infection. As attachment likely requires multiple interactions, the virus is predicted to engage a mixture of gangliosides on the cell surface, depending on lipid composition. While gangliosides are likely entry receptors for BKPyV, the main binding epitope of BKPyV, α2,8-disialic acid, is not only present on gangliosides, but also on glycoproteins. It has been shown for another ganglioside-binding polyomavirus that such sialylated glycoproteins act as decoy receptors [24]. The additional contacts with the left arm of b-series gangliosides therefore may increase BKPyV binding affinity for ganglioside ligands and distinguish those from glycoproteins, which likely would lead the virus along non-infectious entry pathways.

The importance of b-series gangliosides for BKPyV infection may have implications for BKPyV tropism and pathogenesis. Biochemical analyses indicate that the kidney, where BKPyV persists, is rich in diverse sphingolipids and particularly gangliosides. The most abundant gangliosides in adult human kidney are GM3 and GD3, but small amounts of more complex gangliosides were also detected [25], [26]. The relative abundance of simple gangliosides differs between the kidney and the brain, where complex gangliosides are most abundant in adults [27]. Therefore, the differences in affinity toward b-series gangliosides are only one determinant of their usage as receptors *in vivo*, as a lower affinity can be balanced by a greater abundance in the host tissue. Moreover, gangliosides are differentially expressed in cortical tubular, medullary and glomerular tissues of adult human kidney and developmental changes in ganglioside expression have been observed in bovine kidney [26]. Thus, our observation of differing affinities toward b-series gangliosides raises the question whether developmental or drug-induced changes in ganglioside distribution may play a role in BKPyV latency and reactivation.

Structural comparison of BKPyV-GD3 with the closely related SV40-GM1 complex suggested that a protruding lysine at position 68 may prevent BKPyV from binding GM1. To test this hypothesis, we introduced the smaller SV40 residue S68 into BKPyV VP1. This single mutation switched the oligosaccharide specificity of BKPyV from b-series gangliosides to GM1, altering a key attachment property of BKPyV. Known BKPyV VP1 sequences contain lysine, arginine or histidine at position 68. All of these amino acids would block engagement of GM1 but promote or at least tolerate binding of b-series gangliosides. BKPyV strains do not carry a serine at position 68, and this may indicate that recognition of GM1 instead of b-series gangliosides may not be advantageous to the virus in the context of the host organism. Possible explanations could be that GM1 is not very abundant or is differentially localized in human kidneys [25], [26]. There likely exists an evolutionary constraint on BKPyV to bind b-series gangliosides, not GM1, especially as the remainder of the SV40 GM1 binding site is mostly conserved in BKPyV.

We have shown that unlike SV40, the BKPyV K68S mutant is specific for GM1 containing the human sialic acid NeuNAc, and cannot engage its simian counterpart NeuNGc due to steric hindrance. As the WT BKPyV and K68S binding sites for terminal sialic acid are identical, WT BKPyV shares the same sialic acid specificity. The inability of K68S to propagate in simian Vero cells and its ability to attach to and propagate in human HEK cells highlights the importance of sialic acid specificity for viral species tropism. Collectively, our data on BKPyV, JCPyV and SV40 suggest that each virus has adapted to the most prominent sialic acid in its host.

SV40, JCPyV and BKPyV all feature a conserved platform of core residues that allows them to efficiently engage terminal sialic acid in a similar manner. These core residues mediate the vast majority of interactions. However, each virus achieves its distinct receptor specificity with a small number of strategically positioned satellite residues, such as K68 in the case of BKPyV, that form distinct contacts with additional carbohydrate moieties. Thus, these satellite residues define the context in which a terminal sialic acid can be bound, and as demonstrated here they present attractive opportunities for switching receptor specificities.

It is tempting to speculate that at least some members of the polyomavirus family have evolved from an initial sialic-acid binding template through subtle modification of their satellite residues, thereby expanding their host range and tropism. The switching of specificity can occur naturally in viruses, and often triggers altered pathogenicity and species tropism. In many cases, switching is due to exceedingly small changes in the virus capsid structure. Prominent examples include different serotypes of adenoviruses, the canine and feline parvoviruses, as well as avian, swine and human influenza viruses [28], [29], [30]. In many of these cases, however, the molecular functions of these switches, such as how specific mutations alter the interaction with receptors, are not well understood at the atomic level. Switching polyomavirus receptor specificities, as demonstrated here in a first example, may therefore be a useful tool to study parameters that define host receptor recognition, viral uptake, and entry pathways.

## Materials and Methods

### Virus infection

Cells (ATCC, Manassas, VA) were maintained at 37°C in Cellgro Minimum Essential Medium Eagle (MEM) supplemented with 5% heat inactivated fetal bovine serum (Atlanta Biologicals) and penicillin (10,000 U/ml) and streptomycin (10,000 µg/ml) (Gibco). Cells seeded in 24-well dishes were pre-incubated with media, dimethyl sulfoxide (DMSO) or gangliosides GM1, GD2, GD3, GD1b, and GT1b (Matreya) at 0.3–30 µM for 17 h at 37°C. Prior to infection cells were chilled for 20 min at 4°C and washed with 2% MEM. Cells were infected with 8×10^5^ Fluorescent Forming Units (FFU) per ml of BKPyV for 1 h at 37°C. The infectious media was then removed and replaced with fresh growth media. Infection was scored 72 h post infection by staining for VP1 and analyzed by indirect immunofluorescence. Construction of the BKPyV pUC-19 expression plasmid was previously described [20]. BKPyV VP1 mutants were generated by site directed mutagenesis using QuickChange XL (Stratagene, La Jolla, CA). Mutant and WT plasmids were digested with BamHI (Promega). Vero or HEK cells were transfected with (0.5 µg) of linearized mutant or WT BKPyV plasmid DNA using Fugene 6 (Roche).

### Indirect immunofluorescence

To detect expression of viral antigens cells were fixed with 2% paraformaldehyde in phosphate buffered saline (PBS) for 20 min at 25°C and permeabilized with 1% Triton-X 100 in PBS for 15 min at 37°C. Cells were incubated with the primary mouse monoclonal antibody PAb597 (1∶10 [20], or PAb416 (Ab-2) (0.2 mg/ml) (Calbiochem), [31], used at 8 ng/µl to stain for BKPyV T-Ag. After incubation cells were washed with PBS and incubated with Alexa Fluor 488-labeled goat anti-mouse antibody in PBS (Invitrogen).

### Flow cytometry

Vero or HEK cells were incubated with media or 3 µM GM1 for 17–18 h. Cells were washed and suspended in 100 µl (10 µg/mL) (Sigma) of CTX or PBS for 30 min on ice with 10 min agitation. Cells were washed and then incubated with 100 µl of purified wild type or mutant BKPyV VP1 pentamers (100 µg/mL) in PBS on ice for 2 h with 30 min agitations or with PBS alone. Cells were washed and suspended in 100 µl of Penta-His-AlexaFlour 488 conjugated antibody (10 µg/mL) (Qiagen) in PBS on ice for 1 h with 15 min agitations. Cells were washed and fixed in 1% paraformaldehyde and binding analyzed using a BD FACSCanto II flow cytometer (Benton, Dickinson, and Company). Data were analyzed using Flow Jo (Tree Star Inc.) software.

### Recombinant protein expression and purification

We expressed and purified a truncated form of BKPyV VP1 that assembles into pentamers but does not form capsids. DNA coding for amino acids 30–300 of BKPyV VP1 was amplified by PCR and cloned into the pET15b expression vector (Novagen) in frame with an N-terminal hexahistidine tag (His-tag) and a thrombin cleavage site. The protein was overexpressed in *E. coli* BL21(DE3) and purified by nickel affinity chromatography and gel filtration on Superdex-200. For crystallization, the tag was cleaved with thrombin before gel filtration, leaving non-native amino acids GSHM at the N-terminus.

### STD NMR measurements

All NMR spectra were recorded using 3 mm tubes on a Bruker DRX 500 MHz spectrometer fitted with a 5 mm cryogenic probe at 283 K and processed with TOPSPIN 2.0 (Bruker). For all proteins used for STD NMR, two NMR samples were prepared, containing either 1 mM GM1 oligosaccharide (Alexis) or 1 mM GD3 oligosaccharide (Sigma). Protein concentrations were between 19 µM and 22 µM. An additional sample contained 20 µM WT BKPyV VP1 and 1 mM GD1b oligosaccharide (Elicityl, F). Additional protein-free samples were prepared that only contained 1 mM GM1, GD3 or GD1b oligosaccharide. These samples were used to verify that no direct excitation of ligand resonances occurred during STD NMR measurements, and they served as samples for the spectral assignment. 0.1 mM trimethylsilyl propionate was then added to the GD3 sample to allow ^1^H referencing. The buffer used for all NMR measurements contained 20 mM deutero-Tris pH 7.5, 150 mM NaCl, and 20 mM deutero-DTT. Samples were prepared in D_2_O and no additional water suppression was used to avoid affecting the anomeric proton signals. The off- and on-resonance frequencies were set to 80 ppm and 7 ppm, respectively. The total relaxation delay was 4 s. A cascade of 40 Gaussian-shaped pulses with 50 ms duration each, corresponding to a strength of 65 Hz, and a saturation time of 2 s was used for selective excitation. A 10 ms continuous-wave spin lock filter with a strength of 3.7 kHz was employed in order to suppress residual protein signals. 32 k points were collected and zero filling to 64 k data points was employed. Spectra were multiplied with an exponential line broadening factor of 1 Hz prior to Fourier transformation.

To assign oligosaccharide proton resonances, series of 1D ^1^H-TOCSY and COSY spectra as well as ^1^H, ^13^C-HSQC spectra were acquired. Literature values for related oligosaccharides served as additional assignment controls [32], [33], [34], [35]. Assignment of the acetate methyl groups was taken from [34] for GD3 and [32] for GM1.

### Crystallization and structure determination

For crystallization, BKPyV VP1 was supplemented with 20 mM DTT and concentrated to 6.6–7.0 mg/ml. The protein was crystallized at 20°C by sitting drop vapor diffusion against a reservoir of 16–18% PEG 3,350, 0.1 M HEPES pH 7.5 and 0.25 M LiCl (drop size 300 nl protein+300 nl reservoir). Crystals were harvested into reservoir solution containing 14–16% PEG 3,350 and cryoprotected by soaking in harvesting solution supplemented with 30% (v/v) glycerol for 10 s before flash-freezing them in liquid nitrogen. For oligosaccharide complex formation, crystals were soaked in harvesting solution containing 20 mM GD3 oligosaccharide for 15 min before cryoprotection.

Diffraction data were collected at the SLS (Villigen, CH) and processed with xds [36], and the structure was solved by molecular replacement with Phaser [37] using the core of the SV40 VP1 pentamer (3BWQ) as the search model. After rigid body and simulated annealing refinement in Phenix [38], missing parts of the model were built in Coot [39]. Refinement proceeded by alternating rounds of refinement in Refmac5 [40] and model building in Coot. Fivefold non-crystallographic symmetry restraints were used throughout refinement. Oligosaccharide residues were located in weighted 2mFo-DFc and mFo-DFc electron density maps and refined with restraints from the CCP4 monomers library; only the α2,3- and α2,8- glycosidic linkages had to be user-defined. Data collection and refinement statistics are given in Table 1. Coordinates and structure factor amplitudes were deposited with the RCSB data bank (www.rcsb.org) with entry codes 4MJ0 (BKPyV VP1 bound to GD3) and 4MJ1 (unliganded BKPyV VP1). Structure figures were prepared with PyMol (Schrödinger Inc.).

### Molecular modeling

To generate a model for the complex, we first explored the conformational space of GD1b alone using high-temperature molecular dynamics (MD) [41] and subsequently positioned the individual sampled snapshots into the binding site using the α2,8-disialic acid motif of crystal structure as an anchor point. Molecular dynamics simulation of GD1b was performed at 700 K for 100 ns using the MM3 force field as implemented in the TINKER software (http://dasher.wustl.edu/tinker/). Torsion restraints were applied on the ring torsions to avoid inversion of the carbohydrate rings during MD. Snapshots were recorded every 0.5 ps resulting in a conformational ensemble consisting of 200000 frames. Further processing of the data was performed using Conformational Analysis Tools (CAT) (http://www.md-simulations.de/CAT/). Conformational maps were calculated as described [41] in order to check that the accessible conformational space of the glycosidic linkages was sufficiently explored (Supplemental Fig. S1). All snapshots were positioned into the crystal structure using three atoms of residue NeuNAc 4R as an anchor and conformations that result in atom-overlaps with the protein were removed. Additionally two filters were applied on the remaining snapshots that control the position and orientation of residue NeuNAc 3R: Only snapshots were allowed to pass that have the center of the carboxylate and the *N*-acetyl group within 3 Å of the corresponding residue of the crystal structure. Several conformations were manually selected and refined based on 5 ns MD simulations at 300K in explicit water using YASARA [42]. Model coordinates are available from the authors upon request.

### Carbohydrate microarray analyses

Microarrays comprised lipid-linked oligosaccharide probes, neoglycolipids (NGLs) and glycolipids, robotically printed on nitrocellulose-coated glass slides using a non-contact instrument [43], [44]. For the analyses, an array set of 21 ganglioside-related probes (18 sialylated and 3 non-sialylated, in house designation Ganglioside Dose Response Array set 1) was used, in which each probe was arrayed at four levels: 0.3, 0.8, 1.7 and 5.0 fmol/spot. The microarray analyses were performed essentially as described [17]. In brief, microarrays were blocked in 5 mM HEPES (pH 7.4), 150 mM NaCl, 0.3% (v/v) Blocker Casein (Pierce), 0.3% (w/v) bovine serum albumin (Sigma) and 5 mM CaCl_2_ (referred to as HBS-Casein/BSA). WT and mutant BKPyV VP1 were diluted in HBS-Casein/BSA and overlaid at 300 µg/ml and 150 µg/ml, respectively, followed by incubation with mouse monoclonal anti-poly-histidine and biotinylated antimouse IgG antibodies (both from Sigma). SV40 VP1 was tested as a protein-antibody complex that was prepared by preincubating with mouse monoclonal anti-poly-histidine and biotinylated anti-mouse IgG antibodies at a ratio of 4∶2∶1 (by weight) and diluted in HBS-Casein/BSA to provide a final SV40 VP1 concentration of 150 µg/ml. The SV40 and K68S protein samples had been supplemented with DTT to prevent dimerization of pentamers, while WT BKPyV VP1 was analysed without DTT. Binding was detected with Alexa Fluor-647-labelled streptavidin (Molecular Probes). Microarray data analyses were as described [45].

## Supporting Information

Figure S1 **Conformational energy maps for the GD1b oligosaccharide.** The maps show the accessible conformational space of the glycosidic linkages and are calculated based on 200000 snapshots sampled from a 100 ns MD simulation at 700 K using TINKER/MM3 as described in (Frank et al., 2007). Three local energy minima are predicted for the internal NeuNAc-α2,3-Gal linkage.
(PDF)

Figure S2 **Growth of BKPyV K68S in the absence of exogenous GM1.** (A) Vero cells were transfected as previously described, treated with media lacking GM1 ganglioside addition, fixed and stained over 23 days. Viral spread was quantified by scoring for cells expressing T-Ag. (B) HEK cells were transfected as previously described, treated with media lacking GM1 ganglioside addition, fixed and stained over 13 days. Viral spread was quantified as above.
(TIF)

Table S1 **Oligosaccharide probes.** The table lists probes with their sequences included in the ganglioside ‘dose- response’ set 1.
(DOC)
