# Supplementary material for: A Structure-Guided Mutation in the Major Capsid Protein Retargets BK Polyomavirus
Source: PLoS Pathog. 2013 Oct 10;9(10):e1003688. doi: 10.1371/journal.ppat.1003688 (PMC3795024; doi:10.1371/journal.ppat.1003688)
Supplement: Figure S1 — Conformational energy maps for the GD1b oligosaccharide. The maps show the accessible conformational space of the glycosidic linkages and are calculated based on 200000 snapshots sampled from a 100 ns MD simulation at 700 K using TINKER/MM3 as described in (Frank et al., 2007). Three local energy minima are predicted for the internal NeuNAc-α2,3-Gal linkage. (PDF) [file ppat.1003688.s001.pdf]

Neu et al.

**Supplemental Figure S1.**

Conformational energy maps for the GD1b oligosaccharide showing the accessible conformational space of the glycosidic linkages. The maps are calculated based on 200000 snapshots sampled from a 100ns MD simulation at 700 K using TINKER/MM3 as described in (Frank et al., 2007). Three local energy minima are predicted for the internal Neu5Aca(2-3)Gal linkage.

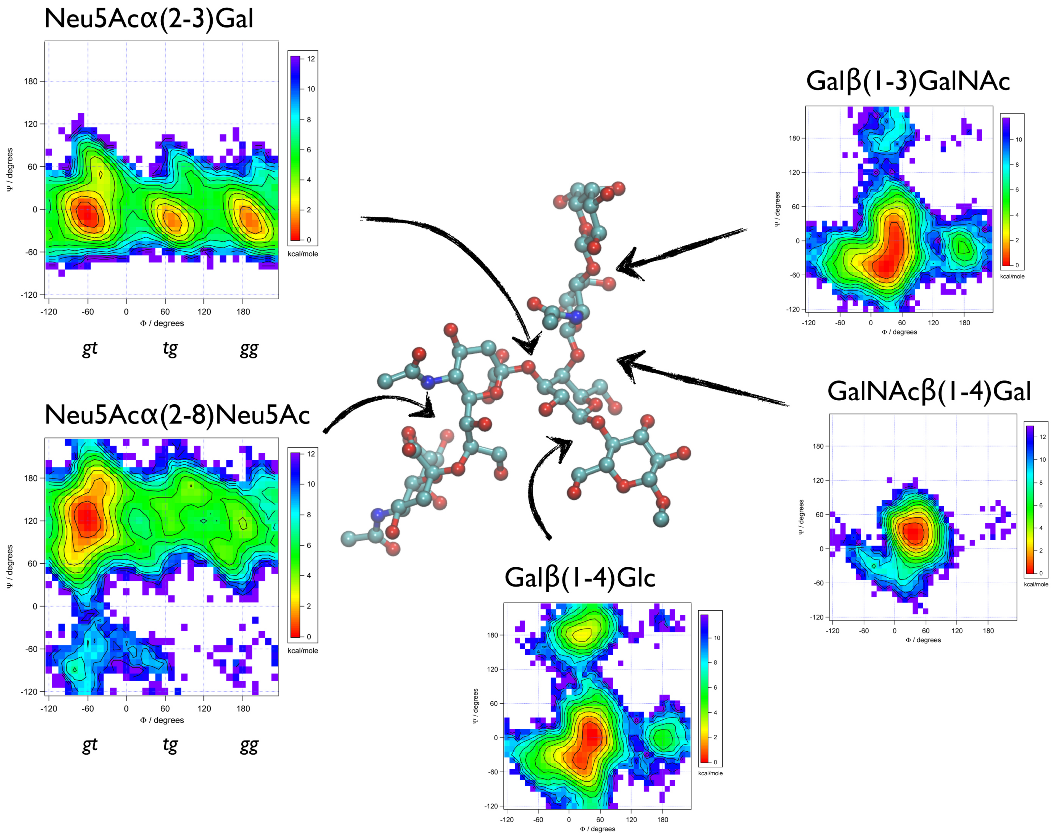


**Reference:**


Frank, M., Lutteke, T., and von der Lieth, C.W. (2007). GlycoMapsDB: a database of the accessible conformational space of glycosidic linkages. Nucl Acid Res *35*, 287-290.
