# Supplementary figures and images for: A Structure-Guided Mutation in the Major Capsid Protein Retargets BK Polyomavirus
Source: PLoS Pathog. 2013 Oct 10;9(10):e1003688. doi: 10.1371/journal.ppat.1003688 (PMC3795024; doi:10.1371/journal.ppat.1003688)

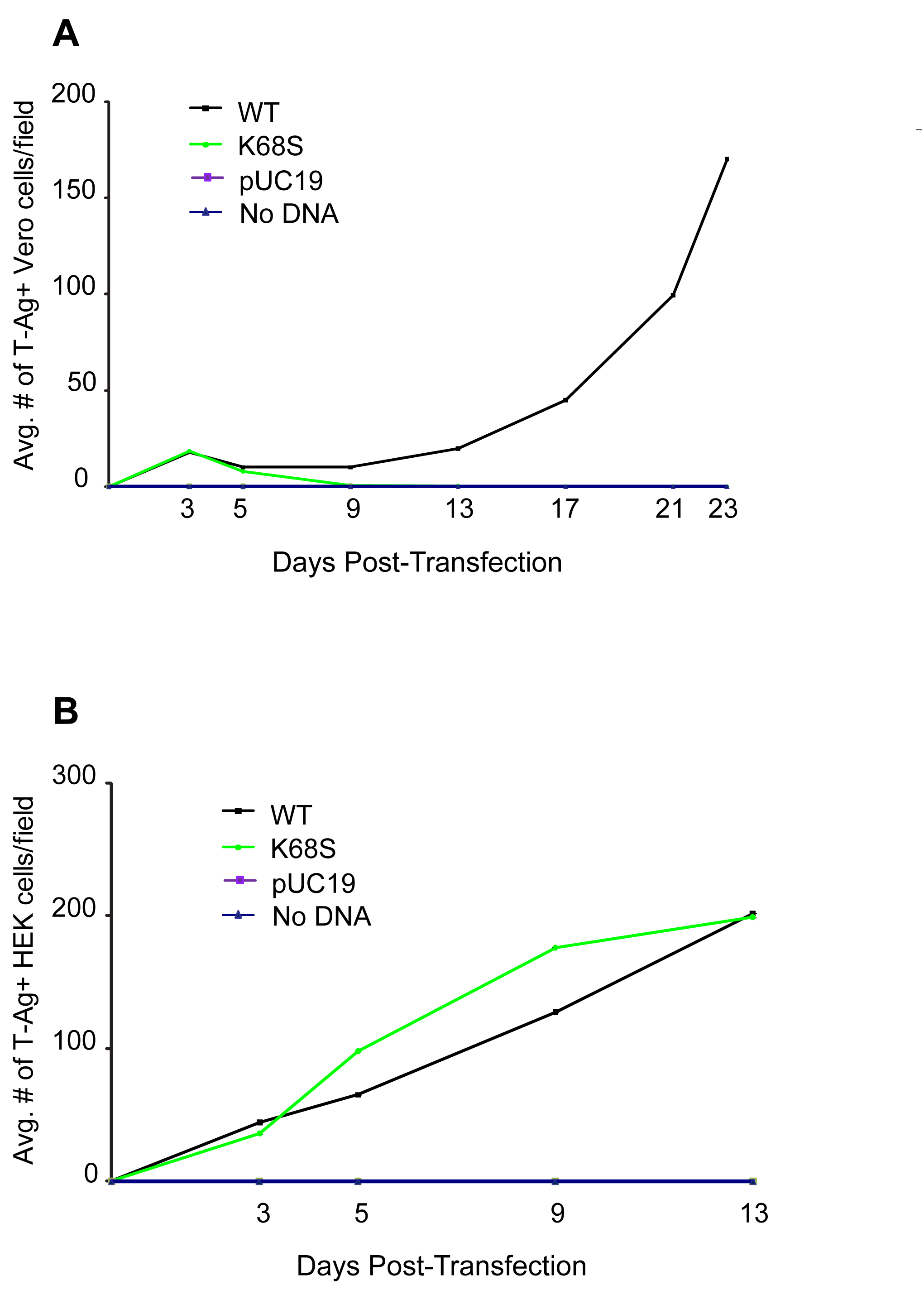

Supplement: Figure S2 — Growth of BKPyV K68S in the absence of exogenous GM1. (A) Vero cells were transfected as previously described, treated with media lacking GM1 ganglioside addition, fixed and stained over 23 days. Viral spread was quantified by scoring for cells expressing T-Ag. (B) HEK cells were transfected as previously described, treated with media lacking GM1 ganglioside addition, fixed and stained over 13 days. Viral spread was quantified as above. (TIF) [file ppat.1003688.s002.tif]
