# Supplementary material for: A Structure-Guided Mutation in the Major Capsid Protein Retargets BK Polyomavirus
Source: PLoS Pathog. 2013 Oct 10;9(10):e1003688. doi: 10.1371/journal.ppat.1003688 (PMC3795024; doi:10.1371/journal.ppat.1003688)
Supplement: Table S1 — Oligosaccharide probes. The table lists probes with their sequences included in the ganglioside ‘dose- response’ set 1. (DOC) [file ppat.1003688.s003.doc]

**Supplemental Table S1. List of probes with their sequences included in the ganglioside ‘dose- response’ set 1.**

| **Position** | **Probe** | **Sequence** |
| --- | --- | --- |
| 1 | GM4 | NeuAcα-3Galβ-Cer |
| 2 | GM3 | NeuAcα-3Galß-4Glcß-Cer |
| 3 | GM3(Gc) | NeuGcα-3Galß-4Glc-Cer |
| 4 | GD3 | NeuAcα-8NeuAcα-3Galß-4Glcß-Cer |
| 5 | Asialo-GM2 | GalNAcβ-4Galβ-4Glcβ-Cer |
| 6 | GM2 | GalNAcβ-4Galβ-4Glcβ-Cer   │   NeuAcα-3 |
| 7 | GD2 | GalNAcß-4Galß-4Glcß-Cer  │ NeuAcα-8NeuAcα-3 |
| 8 | Asialo-GM1 | Galβ-3GalNAcβ-4Galβ-4Glcβ-Cer |
| 9 | Asialo-GM1-Tetra | Galß-3GalNAcß-4Galß-4Glc-DH |
| 10 | GM1 | Galβ-3GalNAcβ-4Galβ-4Glcβ-Cer   │   NeuAcα-3 |
| 11 | GM1-penta | Galβ-3GalNAcβ-4Galβ-4Glc-DH  │   NeuAcα-3 |
| 12 | GM1(Gc) | Galβ-3GalNAcβ-4Galβ-4Glcβ-Cer   │   NeuGcα-3 |
| 13 | GM1(Gc)-penta | Galβ-3GalNAcβ-4Galβ-4Glc-DH  │  NeuGcα-3 |
| 14 | GD1a | NeuAcα-3Galß-3GalNAcß-4Galß-4Glcß-Cer  │  NeuAcα-3 |
| 15 | GD1a-hexa | NeuAcα-3Galß-3GalNAcß-4Galß-4Glc  │  NeuAcα-3 |
| 16 | GD1b | Galß-3GalNAcß-4Galß-4Glcß-Cer  │ NeuAcα-8NeuAcα-3 |
| 17 | GT1a | NeuAcα-8NeuAcα-3Galβ-3GalNAcβ-4Galβ-4Glcβ-Cer  │  NeuAcα-3 |
| 18 | GT1b | NeuAcα-3Galβ-3GalNAcβ-4Galβ-4Glcβ-Cer  │  NeuAcα-8NeuAcα-3 |
| 19 | GQ1b | NeuAcα-8NeuAcα-3Galβ-3GalNAcβ-4Galβ-4Glcβ-Cer  │  NeuAcα-8NeuAcα-3 |
| 20 | LSTc | NeuAcα-6Galβ4-GlcNAcβ3-Galβ4-Glc-DH |
| 21 | LSTa | NeuAcα-3Galß-3GlcNAcß-3Galß-4Glc-DH |

Cer, natural glycolipids with various ceramide moieties; DH, neoglycolipids prepared from reducing oligosaccharides by reductive amination with the amino lipid, 1,2-dihexadecyl-*sn*-glycero-3-phosphoethanolamine.
